# A systematic review on community-based screening of newly arrived migrants in Europe for tuberculosis, human immunodeficiency virus, and hepatitis B and C

**DOI:** 10.1093/eurpub/ckaf234

**Published:** 2026-02-10

**Authors:** Paul W Bird, Daniel Pan, Kentaro Trerattanavong, Christopher A Martin, Christopher Holmes, Laura J Gray, Manish Pareek

**Affiliations:** Department of Microbiology, University Hospitals of Leicester NHS Trust, Leicester, United Kingdom; Department of Respiratory Sciences, University of Leicester, Leicester, United Kingdom; Development Centre for Population Health, University of Leicester, Leicester, United Kingdom; Department of Respiratory Sciences, University of Leicester, Leicester, United Kingdom; Development Centre for Population Health, University of Leicester, Leicester, United Kingdom; Department of Infection and HIV Medicine, University Hospitals of Leicester NHS Trust, Leicester, United Kingdom; Leicester NIHR Biomedical Research Centre, Leicester, United Kingdom; Department of Infectious Diseases and HIV Medicine, University Hospitals of Leicester, NHS Trust, Leicester, United Kingdom; Li Ka Shing Centre for Health Information and Discovery, Oxford Big Data Institute, University of Oxford, Oxford, United Kingdom; WHO Collaborating Centre for Infectious Disease Epidemiology and Control, School of Public Health, Li Ka Sing Faculty of Medicine, University of Hong Kong, Hong Kong, China; Northampton General Hospital NHS Trust, Northampton, United Kingdom; Department of Respiratory Sciences, University of Leicester, Leicester, United Kingdom; Development Centre for Population Health, University of Leicester, Leicester, United Kingdom; Department of Infection and HIV Medicine, University Hospitals of Leicester NHS Trust, Leicester, United Kingdom; Leicester NIHR Biomedical Research Centre, Leicester, United Kingdom; Department of Infectious Diseases and HIV Medicine, University Hospitals of Leicester, NHS Trust, Leicester, United Kingdom; Li Ka Shing Centre for Health Information and Discovery, Oxford Big Data Institute, University of Oxford, Oxford, United Kingdom; Department of Microbiology, University Hospitals of Leicester NHS Trust, Leicester, United Kingdom; Department of Respiratory Sciences, University of Leicester, Leicester, United Kingdom; Leicester NIHR Biomedical Research Centre, Leicester, United Kingdom; Department of Population Health Sciences, University of Leicester, Leicester, United Kingdom; Leicester British Heart Foundation Centre of Research Excellence, Leicester, United Kingdom; Department of Respiratory Sciences, University of Leicester, Leicester, United Kingdom; Development Centre for Population Health, University of Leicester, Leicester, United Kingdom; Department of Infection and HIV Medicine, University Hospitals of Leicester NHS Trust, Leicester, United Kingdom; Leicester NIHR Biomedical Research Centre, Leicester, United Kingdom; Department of Infectious Diseases and HIV Medicine, University Hospitals of Leicester, NHS Trust, Leicester, United Kingdom

## Abstract

Increases in the number of migrants (economic, educational, and involuntary) to Europe from countries with high incidence of communicable diseases [tuberculosis (TB), HIV, and hepatitis B (HBV) and C (HCV)]; has increased the need for cost-effective early disease diagnosis programmes to improve outcomes. We aimed to synthesize and evaluate current literature on community-based screening (CBS) initiatives in Europe, the diseases being screened for, and acceptance when offered. Database search (OVID Medicine, OBIFD EMCAre, and EMBRACE) of studies between January 2000 and January 2024 investigating CBS of newly arrived migrants for TB, HIV, HBV, and HCV in Europe (PROSPERO ID: 542289). Fifteen studies were included TB only (9/15, 60%), blood borne viruses (BBV) (2/15, 14%), and two or more diseases (4/15 26%). Ten (68%) studies were community-based, 3 (16%) in reception centres, 1 (8%) in primary care, and 1 (8%) mixed setting. Five (33%) studies included community leaders/members in recruitment and two (13%) performed follow-up on participants. Screening acceptance ranged from 41% to 100% (TB 41%–100%, BBV 78.5%–100%, TB/BBV 47.3%–100%) and disease prevalence ranged from 0.09% to 45.1% (TB 0.09%–45.1%, BBV 0.2%–8.7%, TB/BBV 3.2%–28.8%). There are few studies investigating CBS of TB or BBV in migrants in Europe, despite a rise in migration over the last decade. This review shows an urgent need for CBS of migrants for multiple infections that includes community members/leaders to improve acceptance rates and reduce disease mobility and mortality in a vulnerable population.

## Introduction

Migration is an important determinant of population change and migration into Europe has increased in recent decades [1]. Newly arrived migrants are defined as individuals living in a country that is not their usual residence having arrived in the past 12 months [2]. Reasons for migration can be economic, educational, or involuntary. Involuntary migrants are often asylum seekers or refugees from countries with a high incidence of communicable diseases, such as tuberculosis (TB) and blood-borne viruses (BBV; HIV, hepatitis B and hepatitis C) [3–5]. The European Centre for Disease Control (ECDC) reported in 2022 that 33.3% of all new TB cases were patients of ‘foreign origin’ and that they presented as the largest group case in several countries include Norway (90.2%), Sweden (83.6%), The UK (77.7%), and Germany (72.4%) [6]. This is concerning, as the European Commission (2024) reported the largest groups receiving residence permits were from countries with high TB incidence, such as India, Morocco, Turkey, Brazil, and Afghanistan. Similarly, the top 15 nationalities of asylum seekers, including Afghanistan, Turkey, Venezuela, Bangladesh, Pakistan, Morocco, and Iraq, were also from high TB burden countries [7].

The WHO European Region 2023–30 TB action plan recommends targeted TB and BBV screening of migrants, which has been adopted by many EU/EEA countries [8]. For example, within the UK, a national latent tuberculosis infection (LTBI) testing and treatment programme was initiated in 2015 and is part of the Tuberculosis Action Plan [8]. The programme aims to test new entrants if they were: born or spend >6 months in a high TB incidence country, entered the UK in the past 5 years, aged between 16 and 35 years, no previous history of TB or latent TB infection or not previously been screened for LTBI [9]. The TB Action Plan is implemented locally through primary or secondary-case based testing/treatment, community-based TB services, or a combination of the three.

In addition to TB, the European Centre for Disease Control (ECDC) reported in 2022 that a significant proportion of member states had outdated (>5 years) guidelines for HIV screening in migrants [10]. The ECDC also reported worse outcomes screening for hepatitis B (HBV) and hepatitis C (HCV), with 61% of countries not reporting HBV diagnoses and 52% not reporting HCV [11].

Currently, public health surveillance relies on healthcare facility data, missing those who are asymptomatic or do not seek care, especially in communities where illnesses like TB or HIV are stigmatized or treated traditionally [12]. A systematic review found that community-based screening (CBS) improves case detection, referrals, follow-up, case management, and health education through community health centres and workers [13].

The ECDC states that screening migrants for TB and BBVs in community settings is more effective for early diagnosis, improves outcomes, and is cost-effective [14]. Community-based screening—defined as systematic screening conducted outside of healthcare facilities among populations at risk [15] or through community-based health workers and centres delivering case detection, referrals, follow-up, and health education—offers a model better suited to underserved groups [12]. Therefore, it is essential to move beyond traditional healthcare systems that fail to meet the needs of those most affected. We conducted a systematic review to examine current CBS initiatives in Europe, the diseases targeted among migrants, and acceptance rates for screening migrants when offered.

## Methods

This study was reported following the Preferred Reporting Items for Systematic Review and Meta-Analysis Protocols (PRISMA-P) guidelines [15]. The review was prospectively registered on PROSPERO (ID: 542289).

### Inclusion/exclusion criteria

We included all peer-reviewed papers on screening for TB and BBV performed in the community in newly arrive migrants, refugees, or asylum seekers in Europe (EU and EEA countries), including the method of diagnosing infection. For this review, community or community-setting is defined as screening taking place outside of traditional healthcare settings, such as secondary care hospitals, but will include primary care as they are based within communities and working through out-reach programmes [12]. Studies that included secondary care facilities were included if they compared results to primary or community-based screening initiatives. We excluded systematic or scoping reviews, academic posters, conference abstracts, clinical trial protocols, non-English language articles, and studies not performed on migrants entering EU/EEA countries.

### Search strategy, data extraction, and synthesis

We searched OVID Medline, OVID-EMCare, and EMBASE to identify eligible papers using key words and MeSH terms with the aid of a Clinical Librarian (LH) for papers published between January 2000 and January 2024; All three databases were searched using the strategy attached in the appendix (Supplementary file S1). Two authors independently reviewed titles and abstracts of all papers based on the inclusion criteria (P.W.B. and K.T.). Reviews were compared and all discordant inclusions or exclusions were screened by a third author (D.P.). All included articles were reviewed by P.W.B. and K.T. and data extracted and collated using Microsoft Excel (Microsoft Excel 365 MSO V.2308). Data extracted included: country the study was conducted in, participant group (migrants, asylum seekers, and/or refugees), study setting (community, reception centre, primary care), community involvement, study start/end date and duration, participant size, age range, sex ratio, race/ethnicity, data collection method, disease(s) screened for, participants screened, results (prevalence), and participants follow-up after screening. Extracted data were reviewed by authors (P.W.B., K.T., and D.P.) to discuss discrepancies and then summarized in tables and figures as well as narratively. Of the included studies, we designated studies as either collecting clinical samples, reviewing medical data, or questionnaire, and calculated acceptance of screening from data extracted (Table 1). Due to the differences in screening tests across studies, it was not possible to perform a meta-analysis of results.

**Table 1.** Definition of review designations.

Review designation	Definition
Collecting clinical samples	A physical sample is taken from the participant as part of the study.
Reviewing medical data	No physical sample taken from participants, but authors given access to previous medical data (e.g. medical notes or screening history)
Questionnaire	Participants completed a questionnaire on their previous screening history
Positive sample	*TB*: A participant positive from tuberculin skin test (TST), culture of organism from sputum, PCR positive by Cepheid GeneXpert MTB/RIF, interferon-gamma release assay (IGRA) positive, or chest X-ray results consistent with TB *HIV*: A participant positive through HIV rapid antibody test from a blood or oral fluid sample, or PCR *HBV/HCV*: A participant positive through serology testing of blood sample or PCR test
Acceptance	Determined by how many participants were included in the study compared to how many consented to screening, access to medic records or completed a questionnaire and calculated a percentage

### Quality assessment

Quality of included studies was assessed based on the Joanna Briggs Institute Critical Appraisal Tool (JBICAT) using the JBICAT Checklist for Systematic Reviews template [16]. Included studies were assessed on whether they included: a clear study design, study aims, participant/population group, study setting, inclusion/exclusion criteria, recruitment method, number of participants, participant age, participant sex, race/ethnicity, screening for multiple infections, acceptance rates, and disease prevalence.

## Results

### Literature search and study overview

**Figure 1.**
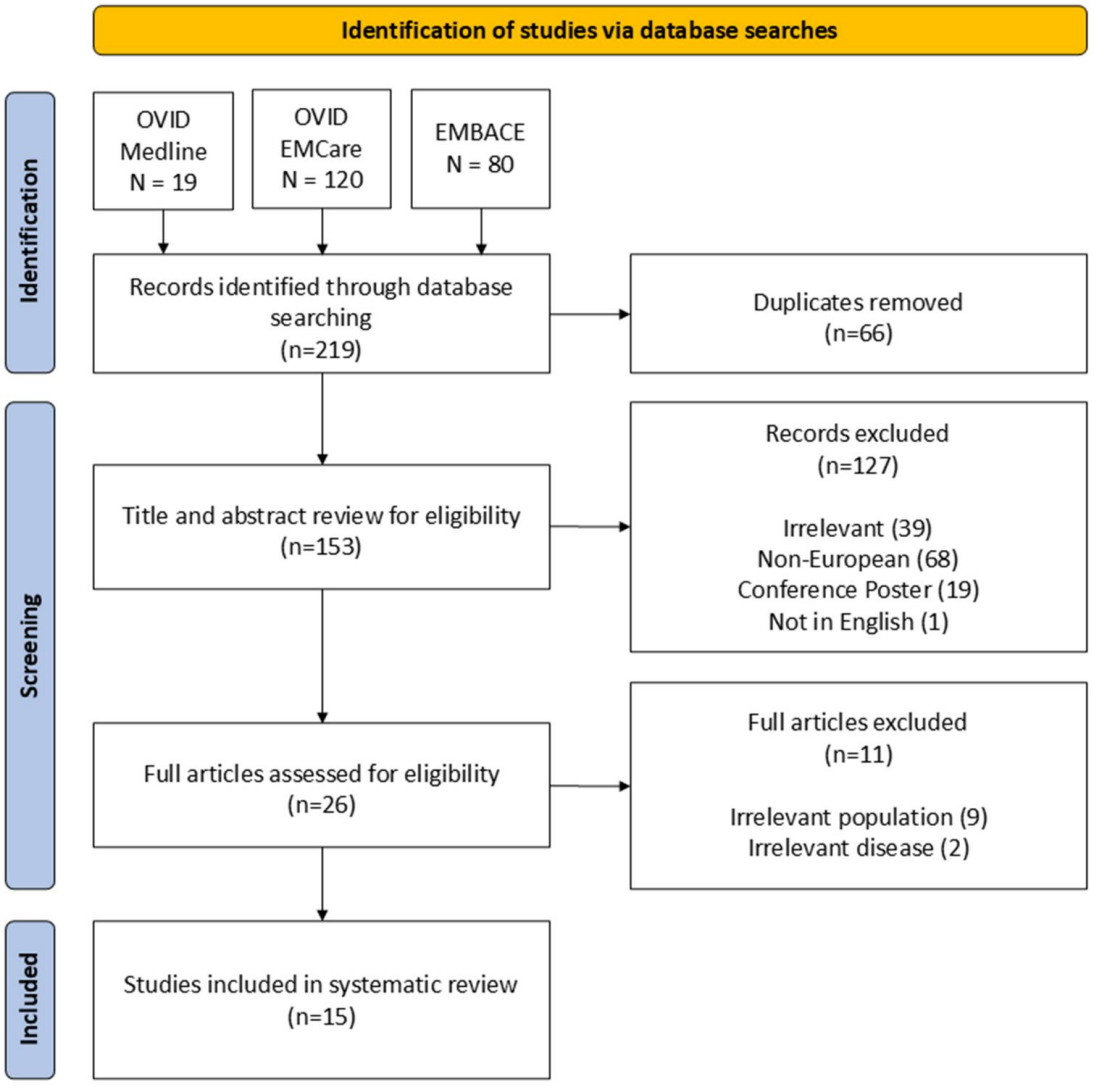
PRISMA diagram depicting the review literature search and screening methodology.

Our search identified 219 unique titles, reducing to 153 once duplicates were removed. After reviewing titles and abstract for eligibility, 25 papers were identified; further reducing to 15 once the papers were read in full (Fig. 1). Across the 15 papers there were 240 223 participants and studies ranged from 1 to 90 months [17–31]. Five studies were conducted in Italy (32%), four from the UK (28%), two from the Netherlands (13%), two from Switzerland (13%), one from Spain (7%), and one from Turkey (7%). Nine studies screened for migrants in general (58%); four screened asylum seekers (28%); one screened refugees (7%), and one screened a mix of migrants and asylum seekers (7%) (Table 2). The majority (9, 58%) focused on TB; two on HIV (13%); one on HBV (7%) [17–27]. Four studies screened for more than one infection (28%) of which one screened for TB, HIV, HBV and HCV [28–31]. Three methods of data collection were used to identify infection; 10 collected clinical samples (65%); four reviewed medical data (28%); and one employed a questionnaire (7%) (Table 2). Of the studies included, only two (13%) performed a follow-up of participants to record treatment adherence and success rate [22, 26]. Data from the 15 papers included in this systematic review have been collated and presented in Table 3.

**Table 2.** Overview of study characteristics.

Study populations (*n* = 15)	
**Population**	Frequency (%)
Asylum seekers only	4 (24%)
Migrants (non-specified)	9 (60%)
Migrants and asylum seekers	1 (8%)
Refugees only	1 (8%)
**Disease included in study**	
Includes tuberculosis only	9 (60%)
Includes HIV only	1 (8%)
Includes HBV only	1 (8%)
Includes HCV only	0 (0%)
Includes two or more disease	4 (24%)
**Data collection**	
Collecting clinical sample	10 (68%)
Questionnaire	1 (8%)
Reviewing medical data	4 (24%)
**Study setting**	
Community setting	10 (68%)
Reception centre	3 (16%)
Primary care	1 (8%)
Community, primary and secondary	1 (8%)

**Table 3.** Summary of studies investigating community-based infectious disease screening in newly arrived migrants in Europe.

Author	Title	Year of publication	Country of study	Participants	Study setting	Community involved in recruitment	Start/end date	Participant size	Age range (years)	Sex ratio	Race/ethnicity	Data collection method	Disease(s) screened for	Participants screened	Results (prevalence)	Follow-up performed
Abubakar *et al.* [17]	Prognostic value of interferon-γ release assays and tuberculin skin test in predicting the development of active tuberculosis (UK PREDICT TB): a prospective cohort study	2018	UK	Migrants	Community setting	Yes	May 2010–June 2015 (61 months)	4749	26–47	Females 2329 (49%), male 2376 (50%), unknown 44 (1%)	Mixed Bangladeshi, Black African, Black Caribbean, Indian, Mixed, Other, Pakistani, White	Questionnaire	Active TB	4749 (100%)	TB 34 (0.7%)	No
van de Berg *et al.* [18]	Evaluation of tuberculosis screening of immigrants in the Netherlands	2017	The Netherlands	Migrants and asylum seekers	Reception centre	N/A	2005–2010 (60 months)	117 389	0–65	Female 64 135 (54.6%), male 53 254 (45.4%)	Mixed Chinese 14 949 (12.7%), Turkish 13 134 (11.2%), Indian 9310 (7.9%), Moroccan 7425 (6.3%), Indonesian 5888 (5%), Other 66 685 (56.9%)	Review medical data	LTBI	117 389 (100%)	LTBI 108 (0.09%)	N/A
Janssens *et al.* [19]	Screening for tuberculosis in an urban shelter for homeless in Switzerland: a prospective study	2017	Switzerland	Asylum seekers	Community setting	N/A	November 2015–March 2016 (6 months)	832	>16	Not specified	Mixed 26%, Romanian 216 (26%), Switzerland 50 (6%), Other 566 (68%)	Reviewing medical data	Active TB	726 acceptance rate (87%)	TB 0	N/A
Laifer *et al.* [20]	TB in a low-incidence country: differences between new immigrants, foreign-born residents, and native residents	2007	Switzerland	Migrants	Community setting	No	January 1997–July 2004 (90 months)	42 601	16–98	Not stated	Not stated	Collecting clinical samples	Active TB	42 601 (100%)	Active TB 161 (0.4%)	No
Lautet *et al.* [21]	National roll-out of latent tuberculosis testing and treatment for new migrants in England: a retrospective evaluation in a high-incidence area	2018	UK	Migrants	Primary care	No	August 2014–August 2015 (12 months)	5591	<16 to >50	Female 2563 (45.8%), male 3028 (54.2%)	Mixed, Majority Indian sub-continent	Collecting clinical samples	Latent TB	2269 (40.6%)	LTBI 11 (0.5%)	No
Spruijt *et al.* [22]	Latent tuberculosis screening and treatment among asylum seekers: a mixed-methods study	2019	The Netherlands	Asylum seekers	Community setting	Yes	November 2016–December 2017 (13 months)	738	18–34	Female 305 (41.3%), male 414 (58.7%)	Eritrean/Ethiopian 515 (69.8%), Other 223 (30.2%)	Collecting clinical samples	Latent TB	719 (97%)	LTBI 205 (28.5%)	Yes
Usdin *et al.* [23]	Latent tuberculous screening of recent migrants attending language classes: A cohort study and cost analysis	2017	UK	Migrants	Community setting	Yes	February 2014–March 2024 (1 month)	440	15–35	Female 234 (53.2%), male 196 (44.5%), not recorded 10 (2.3%)	Not stated	Collecting clinical samples	Active and latent TB	440 (74.8%)	LTBI 71 (16.1%), active TB 2 (0.5%)	No
Villa *et al.* [24]	Tuberculosis and Latent Tuberculosis Infection Screening Among Asylum Seekers in Milan, Italy	2019	Italy	Asylum seekers	Reception centre	No	January 2016–December 2017 (24 months)	6011	10–39	Female 962 (16%), male 5049 (84%)	Mixed North Africa 299 (5.6%), East African 1155 (21.7%), West African 2415 (45.4%), South Asia 975 (18.3%), Other 350 (6.6%), Unknown 130 (2.4%)	Collecting clinical samples	TB (active and latent)	5324 (88.6%)	TST 2403 (45.1%), LTBI 875/1419 (61.7%), active TB 69/1419 (4.9%)	No
Visalli *et al.* [25]	Health conditions of migrants landed in north-eastern Sicily and perception of health risks of the resident population	2020	Italy	Migrants	Community setting	N/A	January 2014–July 2018 (54 months)	39 104	Not stated	Female 5956 (15.3%), male 30 284 (77.4%), children 2864 (7.3%)	Mixed Syrian, Eritrean, Sudan, Nigeria, Bangladesh, Libya, and Egypt	Reviewing medical data	TB	38 608 (100%)	TB 38 (0.1%)	N/A
Scognamiglio *et al.* [26]	HIV rapid testing in community and outreach sites: results of a nationwide demonstration project in Italy	2018	Italy	Migrants	Community setting	Yes	February 2013–July 2013 (6 months)	1147	23–47	531 Male (57.2%), 391 female (42.1%), transgender 4 (0.4%), unknown 2 (0.2%)	Mixed, Italian 86 (9.3%), Foreign 842 (90.7%), Eastern European (35%), North African (18%), Sub-Saharan (18%)	Collecting clinical samples	HIV	928 (80.9%)	HIV 5 (0.5%)	Yes
Vedio *et al.* [27]	Hepatitis B: report of prevalence and access to healthcare among Chinese residents in Sheffield UK	2013	UK	Migrants	Community setting	Yes	September 2009–June 2012 (33 months)	229	15–86	Female 127 (57.5%), male 102 (42.5%)	Chinese 229 (100%)	Collecting clinical samples	HBV	229 (100%)	HBV 20 (8.7%)	No
Henriquez-Camacho *et al.* [28]	Clinicoepidemiological characteristics of viral hepatitis in migrants and travellers of the +Redivi network	2019	Spain	Migrants	Community, primary, and secondary	N/A	January 2009–September 2013 (56 months)	8521	17–50	Not stated	Mixed, North African 276 (3.2%), Sub-Saharan 2202 (25.8%), North America 8 (0.1%), Caribbean 289 (3.4%), South America 4859 (57%), West Asia 54 (0.6%), SC Asia 432 (5%), East Asia 44 (0.5%), Southeast Asia 48 (0.6%), Australasia 0, Europe 305 (3.6%)	Reviewing medical data	HBV + HCV	8,521 (100%)	HBV 450 (5.3%), HCV 112 (1.3%)	N/A
Karaşahin *et al.* [29]	Results of Viral Hepatitis and Human Immunodeficiency Virus Screening in Afghan Irregular Migrants: A Cross sectional Study (2011-2019)	2021	Turkey	Migrants	Community setting	No	January 2011–January 2019 (96 months)	9167	0–86	Female 972 (10.9%), male 8195 (89.1%)	Afghans 9167 (100%)	Collecting clinical samples	HIV + HBV + HCV	7,196 (78.5%)	HIV 16 (0.2%), HBV 505 (7%), HCV 146 (1.6%)	No
Tucco-Tussardi *et al.* [30]	Screening for hepatitis B virus infection among refugees diagnosed with latent tuberculosis in an Italian community	2021	Italy	Asylum seekers	Community setting	No	January 2015–December 2017 (35 months)	2486	20–30	Female 475 (19.1%), male 2011 (80.9%)	Mixed, African 2030 (81.6%), Asian 431 (17.3%), Eastern European 25 (1.1%)	Collecting clinical samples	LTBI + HBV	2,486 (100%)	LTBI 715 (28.8%), HBV 79 (3.2%)	No
Del Pinto *et al.* [31]	Health status of Afro-Asian refugees in an Italian urban area: a cross-sectional monocentric study	2018	Italy	Refugees	Reception centre	N/A	July 2014–December 2014	93	20–34	Males 93 (100%)	Mixed, African 70 (76%), Asian 23 (24%)	Reviewing medical data	Tuberculosis, HIV, and hepatitis B/C	44 (47.3%)	LTBI 8 (18%), HIV 10 (22.7%), HCV 9 (20.4%)	N/A

### Tuberculosis screening

Nine papers focused on TB screening only; Abubakar *et al.* [17], Laifer *et al.* [20], Lautet *et al.* [21], Usdin *et al.* [23], and Visalli *et al.* [25] focused on migrants (55%), Janssens *et al.* [19] Spruijt *et al.* [22] and Villa *et al.* [24] focused on asylum seekers (33%), and van de Berg *et al.* [18] focused on migrants and refugees (12%); six were conducted in community settings (66%), two in reception centres (22%), and one in primary care (12%) (Table 3). The total number of participants were 212 823, with individual study numbers ranging from 440 to 42 000. Five studies collected clinical samples with acceptance rates of 41% to 100% and recruitment ranging from 440 to 42 601 [20, 21, 24, 25]. Lautet *et al.* [21] and Spruijt *et al.* [22] focused on latent TB using interferon-γ release assays (IGRA), while Laifer *et al.* [20] screened exclusively for active TB using chest X-rays and bacterial culture, and Usdin *et al.* [23] and Villa *et al.* [24] screened for active TB using chest X-ray and latent TB using IGRA and tuberculin skin tests (TST). Six studies included participants 16 or younger (66%) and with only Visalli *et al.* [25] not stating the ages [18–21, 23, 24]. Four studies did not collect clinical samples; Abubakar *et al.* [17] collected questionnaire data on active TB, while van de Berg *et al.* [18] reviewed medical data on LTBI, Janssens *et al.* [19] collected medical data on active TB, and Visalli *et al.* [25] reviewed medical data on TB but did not specify active or latent.

Seven studies included participant sex [predominantly male (94 601; 55%)], eight include participant age (ranging from 0 to 98 years), eight include participant ethnicity (six global mix and one European ethnicity), and study lengths ranging from 1 to 90 months [17–25]. Three studies were performed in reception centres when migrants arrived in host countries, with recruitment numbers ranging between 2269 and 117 389 [18, 21, 24]; whereas the remaining six were performed in community settings when migrants had settled with recruitment ranging from 440 to 38 608 [17, 19, 20, 22, 23, 25]. Three of the six studies that involved contact with migrants using community members to aid in participant recruitment, Spruijt *et al.* [22] reported following up patients to monitor adherence to therapy and outcome [17, 22, 23].

Three of the studies, Abubakar *et al.* [17], Janssen *et al.* [19], and Visalli *et al.* [25] screened for or reviewed data on active TB, while van de Berg *et al.* [18], Lautet *et al.* [21] and Spruijt *et al.* [22] screened for LTBI, Usdin *et al.* [23] and Villa *et al.* [24] screened for active and latent TB, and Visalli *et al.* [25] did not state whether active or LTBI. Prevalence of TB (including active and latent) varied between 0.4% to 62% using a combination of TST, IGRA, and Chest X-rays. Studies reviewing medical data had significantly higher numbers of participants, ranging from 832 to 117 389 and higher acceptance rates of 87% to 100%, but reported much lower prevalence of TB between 0% and 0.1% [18, 19, 25]. We observed that studies conducted in community settings (six) and reception centres (two) had greater acceptance rates for screening, between 87% and 100%, compared to 41% in the one primary care study. Of the nine TB studies, only Abubakar *et al.* [17], Spruijt *et al.* [22] and Usdin *et al.* [23] included community members/leaders in recruitment and running of studies. Abubakar *et al.* [17] recruited participants through places of worship (Hindu and Sikh temples, mosques, and churches), local GP surgeries and place of work. Spruijts *et al.* [22] reported that screening in collaboration with asylum seeker centre staff through weekly meetings improved turn out, and facilitated follow-up with participants that had been relocated from the centre [17]. Usdin *et al.* [23] recruited migrants enrolled on English for Speakers of Other Languages (ESOL) courses using teachers to identify migrant students and demonstrated an almost complete follow up for all participant treatment completion [23].

### Blood-borne viruses screening

Four papers investigated BBV; Scognamiglio *et al.* [26] focused solely on HIV, Vedio *et al.* [27] focused solely on HBV, and Henriquez-Camacho *et al.* [28] focused on HBV and HCV, while Karaşahin *et al.* [29] screened for HIV, HBV, and HCV. Scognamiglio *et al.* [26], Vedio *et al.* [27], and Karaşahin *et al.* [29] were based within the community collecting clinical samples, while Henriquez-Camacho *et al.* [28] was based in community, primary, and secondary care settings reviewing medical data (Table 3). Total number of participants were 24 870 ranging between 229 and 8521 participants and acceptance rates between 79% and 100%. All studies included participant age (range 0–86 years), with Scognamiglio *et al.* [26] and Henriquez-Camacho *et al.* [28] screening migrants from multiple ethnic background, while Vedio *et al.* [27] screened Chinese migrants and Karaşahin *et al.* [29] screened Afghans migrants. Participant sex was included by Scognamiglio *et al.* [26] (predominantly male, 57.2%), Vedio *et al.* [27] (predominantly female, 57.5), and Karaşahin *et al.* [29] (predominantly male, 89.1%) and studies ranged from 5 to 104 months [26, 29].

Scognamiglio *et al.* [26] tested for HIV by collecting saliva samples and employing a rapid HIV test (OraQuick Advance Rapid HIV-1/2 Antibody Test; OraSure Technologies Inc.) and reported an acceptance rate of 80.9% from 1147 participants. Vedio *et al.* [27] screened Chinese migrants for HBV only by collecting DBS samples and testing for hepatitis B surface antigen (HBsAg) and hepatitis B total core antibody (HBcAb) employing the ROCHE COBAS 601 automated analyser; reporting an acceptance rate of 100% from 229 participants [27]. Karaşahin *et al.* [29] screened for HIV, HBV (HBsAg, anti-HBs, anti-HBc-IgG), and HCV (anti-HCV), did not include a method and reported an acceptance rate of 78.5% from 9167 participants [29]. Henriquez-Camacho *et al.* [28] performed a review of 14 546 medical records of immigrants to Spain and did not directly recruit participants [28]. Scognamiglio *et al.* [26], Vedio *et al.* [27], and Karaşahin *et al.* [29] all collect clinical samples from participants and reported similar acceptance rates while Vedio *et al.* [27] and Karaşahin *et al.* [29] reported similar prevalence rates for HBV compared to Henriquez-Camacho *et al.* [28] medical record reviews.

### Screening programmes encompassing TB and BBV

Two papers investigated TB and BBV infections. Tucco-Tussardi *et al.* [30] examined LTBI and HBV, while Del Pinto *et al.* [31] examined TB, HIV, HBV, and HCV. Both studies were conducted in Italy; Tucco-Tussardi *et al.* [30] was community based, collected clinical samples, and participants were asylum seekers only; Del Pinto *et al.* [31] reviewed medical data from refugees at reception centres. Community screening (Tucco-Tussardi *et al.* [30]) had a recruitment rate of 100% (*n* = 2486) and reported a prevalence of 29% for TB and 3% for HBV but did not include HIV or HCV. Less than half of refugees consented to access to their medical records (*n* = 44, 47%). Of those that did consent, the prevalence of TB was 18%; HBV 0%; HIV 23% and HCV 20% [30, 31].

Tucco-Tussardi *et al.* [30] focused on HBV in patients previously diagnosed with LTBI, screening for HBsAg and HBcAb and classifying a positive case as participants positive for both markers [30]. Del Pinto *et al.* [31] examined results for TST for TB, with those positive receiving a chest X-ray, HBV was determined through analysis of a serum sample for HBsAg, HCV through HCVaB, and HIV was screened through combined HIV antigen/antibody test for the HIV p24 antigen and relative antibodies using an ELISA technique [31]. Tucco-Tussardi *et al.* [30] explained that TB screening was performed due to the EU/EEA guidance ‘Public health guidance on screening and vaccination for infectious diseases in newly arrived migrants within the EU/EEA’ while BBV screening was justified through high levels of migration into Italy and higher prevalence of BBV in migrants [30].

Screening acceptance by migrants ranged from 40% to 100%; acceptance of screening in the community ranged from 75% to 100%; acceptance of screening within primary or secondary care ranged from 41% to 100% [30, 31].

### Participant follow-up

Only two studies performed follow-up reports, one screening for TB and the other HIV. Both studies were community-based, collected clinical samples, had high participant numbers (719 and 928) and acceptance rates (80.9% and 97%), and involved community members/leaders. One was conducted in the Netherlands and focused on asylum seekers while the other was conducted in Italy and focused on migrants [22, 26].

### Quality assessment

All studies clearly defined how they designed their studies, their target populations (e.g. migrants or refugees), study setting (e.g. primary care or reception centre), number of participants, inclusion/exclusion criteria, and disease prevalence within their cohorts (Supplementary Table S1). The only consistent flaw was a lack of screening for multiple diseases (11/15; 73%) [17–31].

## Discussion

Our review identified three key findings: first, few studies examined community-based screening (CBS) in Europe, despite rising migration and WHO/ECDC recommendations to reach underserved populations; indicating that Europe is falling short of its international TB and BBV screening commitments [8, 9, 32, 33]. Second, although the ECDC advises simultaneous screening for TB and BBVs, only one programme targeted all four infections, with most focusing on a single disease. Third, while the ECDC recommends involving community members or leaders, only five studies did so, despite evidence that their engagement improves participation, case detection, and reduces disease-related stigma.

### Tuberculosis

Analysis of data from TB only studies revealed three key findings. First, studies reviewing existing data showed higher participation and acceptance than those collecting clinical samples, likely due to data-based studies involving migrants already engaging with healthcare, while sample-based studies target newly arrived or healthcare adverse groups [34, 35]. Second, acceptance was higher in community and reception centres (87%–100%, *n* = 6) than in primary care (41%, *n* = 1), suggesting community settings could improve engagement from underserved groups. Usdin *et al.* [23] also found latent TB screening in community settings cost-effective and recommended integrating with primary care [23]. Third, TB prevalence varied across study types and settings, suggesting possible underestimation of true burden. Villa *et al.* [24] reported higher prevalence among asylum seekers than WHO estimates [24], and in this review, asylum seekers showed a tenfold higher prevalence (28%) compared to migrants overall (3%).

### Blood borne viruses

Analysis of BBV data revealed three key findings. First, few studies (*n* = 4) examined community-based BBV screening among migrants in Europe, though three collected clinical samples with high acceptance rates (78.5%–100%) [26–29]. Second, all were conducted in community settings, either alone (*n* = 3) or alongside primary and secondary care (*n* = 1). Third, BBV prevalence was consistently higher than WHO estimates, suggesting underestimation of the true burden; For example, Scognamiglio *et al.* [26] reported 0.5% HIV prevalence in Italy, compared to the national estimate of 0.01% [35].

### TB and blood borne viruses

Two studies investigated TB alongside at least one BBV; both were conducted in Italy, one recruiting asylum seekers and the other refugees [30, 31]. One took place in a community setting without involving community leaders, while the other was conducted in a reception centre. The community-based study achieved notably higher participation (2486 participants) and acceptance (100%) compared to the reception-centre study (44 participants; 47.3%), supporting findings from TB-only studies that community settings are generally preferred over reception or primary care. However, caution is warranted due to the small number of studies and sample sizes. The community study also collected clinical samples—using TST confirmed by IGRA for LTBI and blood samples for anti-HBV antibodies—demonstrating both acceptance of multi-disease screening and the feasibility of including HIV and HCV testing from the same samples [30, 31].

### Screening acceptance

Screening acceptance among migrants, asylum seekers, and refugees was high (average 87%), indicating strong willingness for infectious disease screening [17–31]. Community-based studies showed 19% higher acceptance than non-community approaches, though not statistically significant; nonetheless, evidence supports expanding CBS programmes [27, 36]. Studies highlight that shared language between primary care workers and migrants improves engagement, and link workers can help bridge care gaps between primary care and migrant healthcare users [36]. Implementing CBS in familiar settings such as places of worship or charities can reduce language and cultural barriers, fostering trust and participation. Despite overall high acceptance, only two studies conducted follow-up with participants, with one (Spruijt *et al.* [22]) demonstrating successful community screening among asylum seekers but providing limited follow-up data [22].

### Quality assessment

The majority of studies clearly reported their target population, setting, sample size, and disease prevalence, enabling comparison across healthcare facilities and insights into migrant screening preferences (Supplementary Table S1). However, many lacked clarity on inclusion/exclusion criteria, participant ages, and acceptance rates, outcome of the studies, and few screened for multiple infections. These gaps limit the accuracy and applicability of findings, while single-disease screening reduces efficiency and contradicts WHO and ECDC recommendations [3, 9, 32–34]. In addition, by not reporting on outcome of studies, readers are unable to assess the long-term benefits of screening in these populations.

Our systematic review has several limitations. First, the review was limited to studies written in English leading to the exclusion of four papers. Second, we removed all conference posters and protocols from the review. Third, we only included studies that were performed in Europe as migration pressures vary between global regions by ethnic background and disease.

Several papers noted that community-centred screening and care allows for optimal screening recruitment, diagnosis, and follow-up for conditions not restricted to infectious disease, including but not limited to cardiovascular disease and diabetes mellitus [23–30]. Therefore, future work should focus on strategies to develop a feasible method of engaging migrants in the community to improve health outcomes, for other preventable and detectable health conditions.

Prevalence of diseases were markedly higher than rates recorded by the WHO. Villa *et al.* [24] details significant increases of TB cases in asylum seekers, above WHO estimates, and that this could be due to reactivation of latent TB, contraction of TB during travel, or contraction of TB once migrants have arrived and are detained in reception centres [24]. Future studies focused on longer-term monitoring of migrants for infectious diseases based of their statistically increased risk of diseases has the potential to significantly improve morbidity and mortality rates within migrant communities.

In conclusion, we found few papers investigating CBS for TB and BBV in migrants despite the rise in migration over the last decade. In addition, we found most studies focused on single infections despite WHO and ECDC recommendations for multiple-infection testing, and many studies did not include community members/leaders to improve recruitment and reduce disease associated stigmatization. Future works needs to target multiple organisms in several community-based institutions and include community members/leaders to maximize participation and begin to remove stigma associated with these infections.

## Supplementary Material

ckaf234_Supplementary_Data

## Data Availability

No new datasets were generated or analysed for this study. All data extracted and analysed were obtained from previously published studies, which are fully cited in the reference list. Search strategies are available within the manuscript and appendix (Supplementary file S1. Search strategy). Key points Although the WHO and ECDC recommend screening for multiple infections, the majority of studies in Europe were focused on single diseases. There were limited studies investigating community-based screening of migrants. Community members/leaders were not included in during recruitment regardless of evidence by the ECDC that this increases engagement and acceptant of screening. Further research is required into the feasibility of implementing community-based screening into current public health policy and practice.
