# Supplementary material for: A systematic review on community-based screening of newly arrived migrants in Europe for tuberculosis, human immunodeficiency virus, and hepatitis B and C
Source: Eur J Public Health. 2026 Feb 10;36(2):ckaf234. doi: 10.1093/eurpub/ckaf234 (PMC13148154; doi:10.1093/eurpub/ckaf234)
Supplement: ckaf234_Supplementary_Data [file ckaf234_supplementary_data.docx]

**Appendix:**

**Supplementary File 1. Search strategy.**

Key words and MeSH terms were set as (“migrant” OR “refugee” OR “asylum seeker”) AND (“tuberculosis” OR “TB” OR “human immunodeficiency virus” OR “HIV” OR “hepatitis B” OR “HBV” OR “hepatitis C” OR “HCV”) AND (“screening”)

**Table S1.** Quality assessment of included studies using a 13-item appraisal tool evaluating clarity of study design, aims, population, setting, recruitment, participant characteristics, infection screening scope, acceptance rates, and reported disease prevalence.

| **Author** | **Study design explained clearly** | **Study aims explained clearly?** | **Participant/population group clearly identified?** | **Study setting clear** | **Inclusion/exclusion criteria included?** | **Recruitment method explained?** | **No. of participants included** | **Participant ages (including ratios)?** | **Participant sex**  **(Including ratios)** | **Race/Ethnicity (including ratios)** | **Screened for multiple infections** | **Acceptance rates?** | **Disease prevalence included?** |
| --- | --- | --- | --- | --- | --- | --- | --- | --- | --- | --- | --- | --- | --- |
| Abukakar et al. | *✓* | *✓* | *✓* | *✓* | *✓* | *✓* | *✓* | *✓* | *✓* | *✓* | × | *✓* | *✓* |
| van de Berg et al. | *✓* | × | *✓* | *✓* | *✓* | *✓* | *✓* | *✓* | *✓* | × | × | *✓* | *✓* |
| Janssens et al. | *✓* | *✓* | *✓* | *✓* | *✓* | *✓* | *✓* | *✓* | × | *✓* | × | *✓* | *✓* |
| Laifer et al. | *✓* | *✓* | *✓* | *✓* | *✓* | *✓* | *✓* | *✓* | × | *✓* | × | *✓* | *✓* |
| Loutet et al. | *✓* | *✓* | *✓* | *✓* | *✓* | *✓* | *✓* | *✓* | *✓* | *✓* | × | *✓* | *✓* |
| Spruijt et al. | *✓* | *✓* | *✓* | *✓* | *✓* | *✓* | *✓* | *✓* | *✓* | *✓* | × | *✓* | *✓* |
| Usdin et al. | *✓* | *✓* | *✓* | *✓* | *✓* | *✓* | *✓* | *✓* | *✓* | × | × | *✓* | *✓* |
| Villa et al. | *✓* | *✓* | *✓* | *✓* | *✓* | *✓* | *✓* | *✓* | *✓* | *✓* | × | *✓* | *✓* |
| Visalli et al. | *✓* | *✓* | *✓* | *✓* | *✓* | *✓* | *✓* | × | *✓* | *✓* | × | *✓* | *✓* |
| Scognamiglio et al. | *✓* | *✓* | *✓* | *✓* | *✓* | *✓* | *✓* | *✓* | *✓* | *✓* | × | *✓* | *✓* |
| Vedio et al. | *✓* | *✓* | *✓* | *✓* | *✓* | *✓* | *✓* | *✓* | *✓* | *✓* | × | *✓* | *✓* |
| Henriquez-Camacho et al. | *✓* | *✓* | *✓* | *✓* | × | *✓* | *✓* | *✓* | × | *✓* | *✓* | *✓* | *✓* |
| Karaşahin et al. | *✓* | *✓* | *✓* | *✓* | *✓* | × | *✓* | × | *✓* | *✓* | *✓* | *✓* | *✓* |
| Tucco-Tussardi et al. | *✓* | *✓* | *✓* | *✓* | *✓* | *✓* | *✓* | *✓* | *✓* | *✓* | *✓* | *✓* | *✓* |
| Del Pinto et al. | *✓* | *✓* | *✓* | *✓* | *✓* | *✓* | *✓* | *✓* | *✓* | *✓* | *✓* | *✓* | *✓* |
